# Bioactives derived from Brazilian native flora with antimicrobial and anticancer activity

**DOI:** 10.1186/s12906-025-04787-0

**Published:** 2025-03-11

**Authors:** Daniele Cristina Vitorelli-Venancio, Rosemary Matias, Amanda Rodrigues Ganassin, Fabio Antonio Venancio, Renata Trentin Perdomo, Giovana Bicudo Gomes, Angela Kwiatkowski, João Víctor de Andrade dos Santos, Marilene Rodrigues Chang

**Affiliations:** 1 Programa de Pós-Graduação em Doenças Infecciosas e Parasitárias, Universidade Federal de Mato Grosso do Sul, Campo Grande, MS Brazil; 2 Faculdade de Medicina, Centro Universitário de Adamantina, Adamantina, SP Brazil; 3 Programa de Pós-Graduação em Meio Ambiente e Desenvolvimento Regional, Universidade Anhanguera– Uniderp, Campo Grande, MS Brazil; 4 Programa de Pós-Graduação em Ciências Farmacêuticas, Universidade Federal de Mato Grosso do Sul, Campo Grande, MS Brazil; 5 Instituto Federal de Educação, Ciência e Tecnologia de Mato Grosso do Sul, Coxim, MS Brasil; 6 Programa de Pós-Graduação em Ciências da Saúde, Universidade Federal da Grande Dourados, Dourados, MS Brazil

**Keywords:** Antimicrobial, Antiproliferative effects, Antioxidant, Plant extracts, Brazilian cerrado

## Abstract

**Background:**

The development of new drugs that act against multidrug-resistant microorganisms and malignant tumors is necessary owing to the limited therapeutic options and high mortality rates associated with these pathologies. In this study, we evaluated the phytochemical groups present in seven plants from the Brazilian Cerrado even as their antioxidant, antiproliferative and antimicrobial activities.

**Methods:**

The extracts were obtained by the maceration technique and secondary metabolites were determined by phytochemical analysis. The antioxidant activity was assessed by the DPPH (2,2-diphenyl-1-picrylhydrazyl) free radical scavenging method. The antiproliferative activity of the extracts was assessed using human breast, kidney, and liver neoplastic cells. Cytotoxicity was evaluated in a non-neoplastic cell line — NIH/3T3. The antimicrobial activity of the plant extracts against resistant bacteria and yeasts was determined using disk diffusion assays, and the minimum inhibitory concentration (MIC) was determined by the broth microdilution technique.

**Results:**

Phytochemical analysis revealed the presence of phenolic compounds, flavonoids, steroids, tannins, and saponins in all of the extracts, with *Smilax fluminensis* showing the highest levels of phenolic compounds and flavonoids. All tested extracts exhibited antioxidant activity above 50%, notably *Tapiria obtusa* (82.36 ± 0.44). The *T. obtusa* extract showed potent antiproliferative activity against the 786-0 cell line (GI_50_ 10.16 ± 2.33 µg/mL) and a significantly greater SI (SI = 24.61) than the control (SI = 3.23, doxorubicin), indicating its selective cytotoxicity against cancer cells and its potential as a therapeutic agent against renal cancer. No cytotoxicity was observed in non-tumor cells. Extracts of *S. fluminensis* leaves showed fungicidal effects on *Candida glabrata* (MIC = 500 µg/mL). This study is the first to demonstrate the antibacterial activity of *T. obtusa* leaf ethanolic extract against MRSA (MIC = 1,000 µg/mL).

**Conclusions:**

The ethanolic extract of *T. obtusa* demonstrated antioxidant activity, antiproliferative effects against the 786-0 cell line, and antibacterial activity against MRSA. The ethanolic extract of *S. fluminensis* leaves exhibited a fungicidal effect against *C. glabrata*. These findings may pave the way for more effective and safer treatments for managing oncological and infectious diseases.

**Supplementary Information:**

The online version contains supplementary material available at 10.1186/s12906-025-04787-0.

## Background

The discovery and characterization of bioactive compounds from plants are fundamental for the development of new drugs [1, 2]. Studies show that certain compounds exhibit a variety of properties, such as antimicrobial, antiviral, anti-inflammatory, antioxidant, antiprotozoal, and anticancer activities [1, 3, 4].

The development of new drugs that act against multidrug-resistant microorganisms and malignant tumors has been stimulated by the limited therapeutic options and high mortality rates associated with these pathologies [5–7].

There is an urgent need for new therapeutic alternatives for infections caused by multidrug-resistant pathogens, such as methicillin-resistant *Staphylococcus aureus* (MRSA), carbapenemase-producing *Klebsiella pneumoniae* (KP-KPC), and some *Candida* species [6, 8]. In 2019, approximately 1.27 million deaths were attributed to infections caused by resistant pathogens [9].

Cancer is one of the leading causes of mortality worldwide, with approximately 9.7 million deaths in 2022 and an estimated 20 million new cases annually [10]. Breast cancer is the most prevalent type among women and exhibits significant variation in treatment responsiveness [10]. Renal cancer, also with high incidence, faces therapeutic challenges due to resistance to certain conventional chemotherapies [11]. Liver cancer is the third most common and is known for its aggressiveness and frequent late-stage diagnosis, which complicates treatment and reduces survival rates [10, 12].

There are currently many anticancer drugs, however, many of them are accompanied by serious side effects. Advanced therapeutic approaches include targeted therapy, nanomedicine, immunotherapy, gene therapy, and the use of natural products [13]. Paclitaxel, vinblastine, and etoposide are examples of anticancer compounds plant-derived [14–16]. There is evidence that natural agents may treat cancer with fewer adverse effects, reinforcing the importance of natural products in oncology [13].

Natural antioxidants, such as alkaloids, flavonoids, carotenoids, vitamins, berberine, quercetin, and curcumin have demonstrated chemopreventive and therapeutic potential in both in vitro and in vivo cancer models [17, 18]. These compounds protect the body from oxidative stress, neutralize free radicals, and modulate oxidative stress pathways, which are significant contributors to cancer progression and chronic diseases [18–20].

In this scenario, due to its rich biodiversity, the Brazilian Cerrado biome represents an important source of natural resources for the discovery of bioactive compounds that could aid in the development of new medicines [21].

The Cerrado is the second-largest biome in Brazil and harbors vegetation with origins dating back to prehistoric times. Studies suggest that its vegetation structure varies according to altitude and climatic barriers, contributing to the biome’s ecological complexity. The vast and diverse flora supports an estimated 12,600 plant species, many of which hold significant cultural and medicinal value and are utilized by local traditional communities [22, 23].

Previous studies have shown that in folk medicine, the plant *Equisetum pyramidale* (*E. pyramidale*) is used as a remineralizing, diuretic, and anti-inflammatory agent [24]; *Porophyllum ruderale* (*P. ruderale*) is utilized for wound healing as an analgesic, anti-inflammatory agent, antibacterial agent, and fungicide [25, 26]; *Pouteria ramiflora* (*P. ramiflora*) is employed in the treatment of worm infections, diarrhea, nausea, vomiting, pain, and inflammation [27, 28]; *Smilax fluminensis* (*S. fluminensi*s) is used as a diuretic and for treating syphilis, rheumatism, and skin conditions [29], and *Tapirira obtusa* (*T. obtusa*) is utilized for treating skin diseases and syphilis [30].

Considering that the antimicrobial and anticarcinogenic activities of the extracts of these five plants have been underexplored, in this study, we identified phytochemical groups present in the ethanolic extracts of the aforementioned plant species from the Brazilian Cerrado and evaluated their antioxidant, antiproliferative, cytotoxic, and antimicrobial properties.

## Methods

### Collection and identification of plant species

**Fig. 1:**
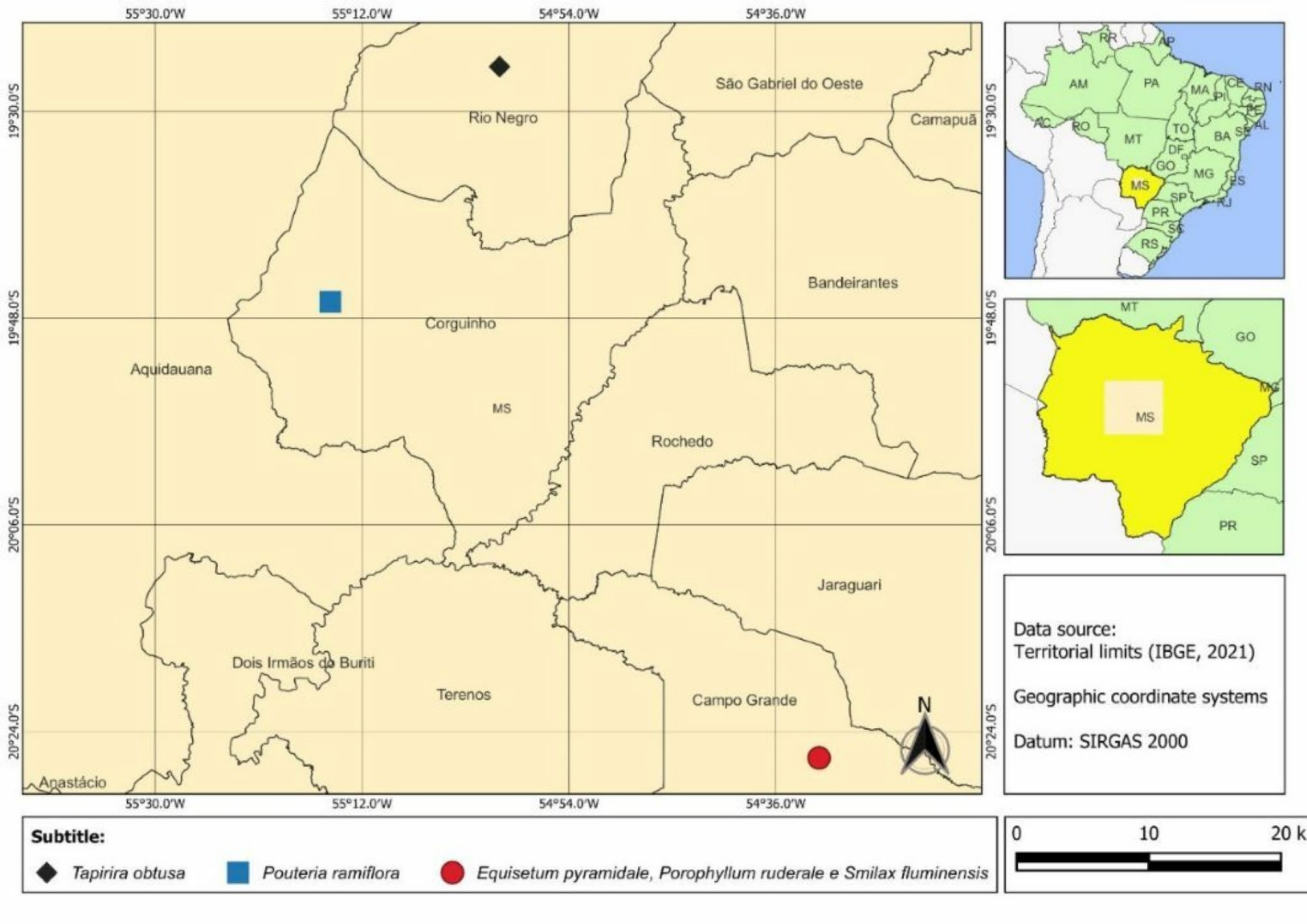
Collection sites of *Equisetum pyramidale*,* Porophyllum ruderale*,* Smilax fluminensis*,* Pouteria ramiflora* and *Tapirira obtusa* from Brazil. Note: The abbreviations shown on the map correspond to the states of Brazil. MS = Mato Grosso do Sul

In this study, we used the leaves and stems of *Equisetum pyramidale* Golden and *Porophyllum ruderale* Jacq., as well as the leaves of *Smilax fluminensis* Steud., *Pouteria ramiflora* (Mart.) Radlk. and *Tapirira obtusa* (Benth.) J.D. Mitch. The plant materials of these five species were collected from the Cerrado biome, Brazil (Fig. 1) at different times, taking into account the specific characteristics of each plant to ensure adequate levels of active compounds, which are generally most abundant before or after flowering and fruiting [31, 32]. *Equisetum pyramidale*, *P. ruderale*, and *T. obtusa* were collected during the Brazilian summer, while *S. fluminensis* and *P. ramiflora* were collected in the winter. Botanical identification was performed by Professor Ademir Kleber Morbeck de Oliveira. Voucher specimens have been prepared and deposited at the University Anhanguera – Uniderp Herbarium, Campo Grande, MS, Brazil. The voucher numbers are as follows: *E. pyramidale* 2214, *P. ruderale* 8021, *S. fluminensis* 8569, *P. ramiflora* 7829, and *T. obtusa* 8439. Additionally, authorization for access to the genetic heritage was obtained from the Genetic Heritage Management Council (CGEN) under registration nº 010579/2013-3.

### Preparation of plant extracts

Following the exclusion of old or damaged leaves, the botanical material were dried in an air-circulating oven (Model MA035/5, Marconi, Piracicaba, Brazil) at 45 ± 1 °C, ground, and subjected to ethanol extraction using an ultrasonic bath for 60 min, followed by static maceration for 24 h. This procedure was repeated for seven days. The crude extract was obtained using a rotary evaporator (Tecnal, MA120, Piracicaba, Brazil) at 55 ± 1 °C [26], with the remaining traces of solvent removed under reduced pressure in a desiccator over silica gel for five days [33]. All the produced extracts were stored in a refrigerator at temperatures between 2 and 8 °C to avoid the degradation of any heat-sensitive compounds.

### Phytochemical screening

The extraction of the crude extract of each plant was done with ethanol (20% dilution) and the identification of phenolic compounds, flavonoids, tannins, coumarins, steroids, triterpenes and thiophenols by classical phytochemical procedures [26].

The saponin content was determined using 1 g of dry plant powder, and foam formation was observed [27]. Analyses were performed in triplicate. The results were compared with those of the control sample by observing the color alteration, precipitation or foam formation. The results were classified as strongly positive with high intensity (+++), markedly positive (++±), positive (++), moderately positive (+±), slightly positive (+), partial (±), or negative (-), which corresponds to frequencies of 100%, 75%, 50%, 25%, 10%, 5%, and 0%, respectively [33].

To confirm the presence of the characteristic chemical groups, each crude extract was diluted in methanol (1.0 mg/1.0 mL) and analyzed with a spectrophotometer (Femto™, 800XI, Brazil) [34]. Maximum absorbance measurements (λmax, 200 to 800 nm) were performed in triplicate.

### Quantification of total phenols and flavonoids

Total phenols were determined using the Folin-Ciocalteu method, using gallic acid as a standard at concentrations of 10, 50, 100, 150, 300, and 400 µg/mL [24].

Flavonoids were evaluated by the aluminum chloride method, and quercetin was used as a standard at concentrations of 6, 8, 10, 12, 16, and 20 µg/mL [24]. Both analyses were performed in triplicate.

### Antioxidant assay

Antioxidant activity was evaluated by the DPPH (2,2 diphenyl-1-picrylhydrazyl) free radical scavenging method according to Mensor et al. [35]. Each extract was solubilized in absolute ethanol to a final concentration of 50 mg/mL. Ascorbic acid (Synth™, Diadema, Brazil), a naturally occurring antioxidant, was used as a positive control. Tests were performed in triplicate.

The ability to scavenge DPPH radical was expressed as the percentage inhibition and calculated using the following equation: AA% = 100 – [(Aa – Ab) × 100]/Ac, where Aa is the absorbance of the sample, Ab is the absorbance of the blank, and Ac is the absorbance of the control, as previously described [35].

### Antiproliferative activity assay

The evaluation of antiproliferative activity was performed using sulforhodamine B (SRB) dye, which is based on the affinity of this compound for basic proteins present in intact cells and is fixed by trichloroacetic acid (TCA) [36]. The plant extracts were tested on four human neoplastic cell lines: MDA-MB231 (ATCC-HTB-26; multidrug resistant breast/triple negative cell), MCF-7 (ATCC-HTB-22; hormone receptor-positive breast), 786-0 (kidney), and HepG2 (liver). A non-neoplastic cell line, NIH/3T3 (ATCC CRL-1658; standardized murine fibroblast by OECD – Organization for Economic Cooperation and Development), was also used to assess cytotoxicity.

Cells were seeded into 96-well plates. Cells without treatment (T0) were read after 24 h of incubation. After this period, the cells were treated with plant extracts at concentrations of 0.25, 2, 5, 25, and 250 µg/mL and incubated for 48 h. Doxorubicin was used as a positive control and TCA was used to fix the cells for 30 min. Afterwards, 50 µL of SRB was added to stain the treated cells. The assay was performed in triplicate.

The percentage of cell growth for each test sample was calculated using SoftMax Pro 6.3. Antiproliferative activity, expressed as GI_50_ (the concentration required to inhibit 50% of cell growth), was determined through nonlinear regression analysis with the ORIGIN 6.0 software, following the formulas described by Monks [37].

For the analysis of the results, we considered that extracts with GI_50_ ≤ 30 µg/mL exhibit potent antineoplastic activity, while GI_50_ values between 30 and 100 µg/mL indicate moderate activity, and those with GI_50_ values > 100 µg/mL reflect weak activity [38–40].

### Selectivity index

The Selectivity Index (SI) is a ratio that measures how selective a compound is against neoplastic cells without impairing the viability of normal cell lines. For the calculation of the SI, the GI_50_ value of the non-neoplastic cells (NIH/3T3) was divided by the GI_50_ value of the neoplastic cell lines. An SI value equal to 2.0 or greater was considered significant [41].

### Antimicrobial susceptibility tests

#### Microorganisms

The antimicrobial activity of the extracts was assessed against human pathogens, including *C. albicans* (ATCC 90028), *C. parapsilosis* (ATCC 22019), and *C. krusei* (ATCC 6258), currently known as *Pichia kudriavzevii*,* C. glabrata* (ATCC 2001), currently known as *Nakaseomyces glabrata*, and resistant bacteria from clinical samples: methicillin-resistant *Staphylococcus aureus -* MRSA (S144), and *Klebsiella pneumoniae -* KP-KPC (S61) isolated in a University Hospital. All the strains we stored − 20 ºC.

#### Disk diffusion method

A stock solution of the crude extract was prepared by dissolving 0.1 g of the extract in 100 mL of distilled water to produce a final concentration of 100,000 µg/mL. The stock solution was then diluted to final concentrations of 25,000 µg/mL and 100,000 µg/mL [adapted from 42]. Sterile paper disks with a diameter of 6 mm were impregnated with 20 µL [42] of the diluted extract at concentrations of 500 µg/disc and 2,000 µg/disc. Subsequently, they were dried in a microbiological safety cabinet for 10 min.

The test was conducted following the methodology of the Clinical Laboratory Standards Institute [43], with adaptations for natural products. Chloramphenicol (30 µg/disc – CECON™ São Paulo, Brazil) and fluconazole (25 µg/disc – CECON™ São Paulo, Brazil) were used as quality controls, and filter paper discs with sterile water were used as negative controls.

The plates were incubated at 35° ± 2 °C for 24 h. The sensitivities of the microorganism species to the plant extracts were determined by measuring the sizes of the inhibitory zones on the agar surface around the disks. Values < 8 mm were considered inactive against microorganisms [44]. The tests were performed in duplicate.

#### Broth microdilution method

The minimum inhibitory concentration (MIC) was determined using the broth microdilution technique adapted from CLSI [45]. A suspension of each microorganism was made in sterile saline (0.85%) with turbidity equivalent to the 0.5 McFarland standard and diluted in the recommended culture media [45, 46]. A stock solution of each plant extract was prepared by dissolving 4 mg of the extract in 1 mL of distilled water to produce a final concentration of 4,000 µg/mL. Then, 100 µL of each extract were added to the wells of a 96-well microtiter plate and serially diluted to obtain concentrations ranging from 2,000 to 3.91 µg/mL. Finally, each well received 100 µL of the microbial suspension. Fluconazole (Sigma™, Saint Louis, USA) and ertapenem (Sigma™, Saint Louis, USA) were used as quality controls at concentrations ranging from 64 to 0.125 µg/mL and 32 to 0.062 µg/mL, respectively. As a negative growth control, we used only the culture medium (without the inoculum), specifically, Roswell Park Memorial Institute (RPMI) 1640 medium for yeasts and cation-adjusted Mueller–Hinton broth for bacteria, and as positive growth control, medium plus microorganism. The plates were incubated at 35 ± 2 °C for 24 h. The tests were performed in duplicate.

After the incubation period, the reading was performed visually with the aid of a concave mirror, and the microbial growth of the positive control was compared with the microbial growth under the effects of antimicrobials and plant extracts. For the interpretation of the results, the MIC was considered the lowest concentration capable of inhibiting 100% of microbial growth.

#### Determination of minimum bactericidal concentration (MBC) and minimum fungicidal concentration (MFC)

From the wells where no microbial growth was observed, a 10 µL aliquot was taken and inoculated onto Petri dishes containing Mueller–Hinton agar (MHA) for bacteria and Sabouraud dextrose agar (SDA) for yeasts and incubated at 35 ± 2 °C.

After 24 h, the plates were analyzed, and the MBC and MFC were defined as the lowest extract concentration at which no microbial growth was observed on the agar surface [47].

### Statistical analysis

The descriptive analysis and the characterization of the findings were performed based on the frequency distribution of the selected variables and the calculation of the mean and standard deviation. For analysis of the antioxidant activity and dosage of phenolic compounds in the samples, analysis of variance (ANOVA) was used to compare the groups of samples. To determine which groups were significantly different, the Tukey test was used.

For all analyses, a significance level of 0.05 or less was adopted. Statistical analysis was performed using R (version 4.3.1) via the RStudio interface [48]. The ggplot2 R package was used to construct the graphs [49].

## Results

### Phytochemical analysis

**Fig. 2:**
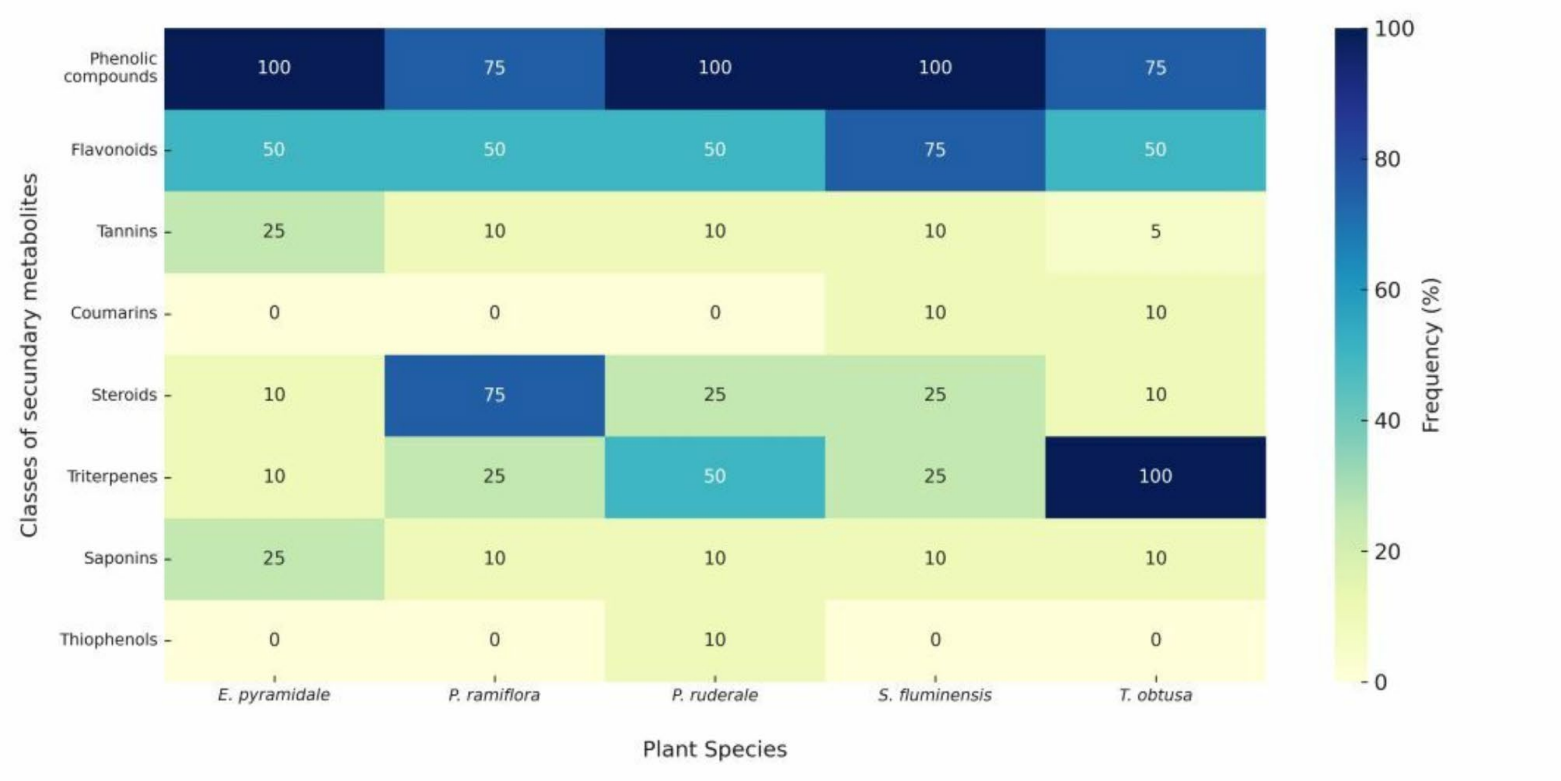
Heatmap of secondary metabolites found in ethanol extracts of plants from the Cerrado biome, Brazil. Note: Frequency of secondary metabolite classes: strongly positive (100%), followed by marked positive (75%), positive (50%), moderately positive (25%), slight positive (10%), partial (5%) and negative (0%)

Phytochemical analyses of the ethanolic extracts indicated the presence of phenolic compounds, flavonoids, tannins, steroids, triterpenes, and saponins in all of the plant species, with a predominance of phenolic compounds and flavonoids (Fig. 2). *Smilax fluminensis*,* P. ruderale*, and *T. obtusa* exhibited the greatest diversity in secondary metabolite classes (seven classes), followed by *E. pyramidale* and *P. ramiflora* (six classes).

The specific absorptions recorded for each sample were as follows: 270, 326, 335, and 368 nm for *E. pyramidale*; 280, 330, 350, and 417 nm for *P. ramiflora*; 230, 260, 335, and 390 nm for *P. ruderale*; 290, 330, and 380 nm for *S. fluminensis*; and 250, 320, 390, and 420 nm for *T. obtusa.*

The confirmation of the major classes observed in the UV-visible spectra was based on the maximum absorption values (λ_max_) identified in each sample. The absorptions in the range of 320 to 390 nm, present in all samples, are characteristic of flavonoids. In contrast, absorptions between 240 and 290 nm indicate the presence of phenolic compounds. Steroids and triterpenes exhibit absorption bands in the region of 410 to 440 nm. Additionally, the band observed at 230 nm in the *P. ruderale* sample suggests the presence of thiophenols.

### Quantification of phenolic compounds and flavonoids

**Fig. 3:**
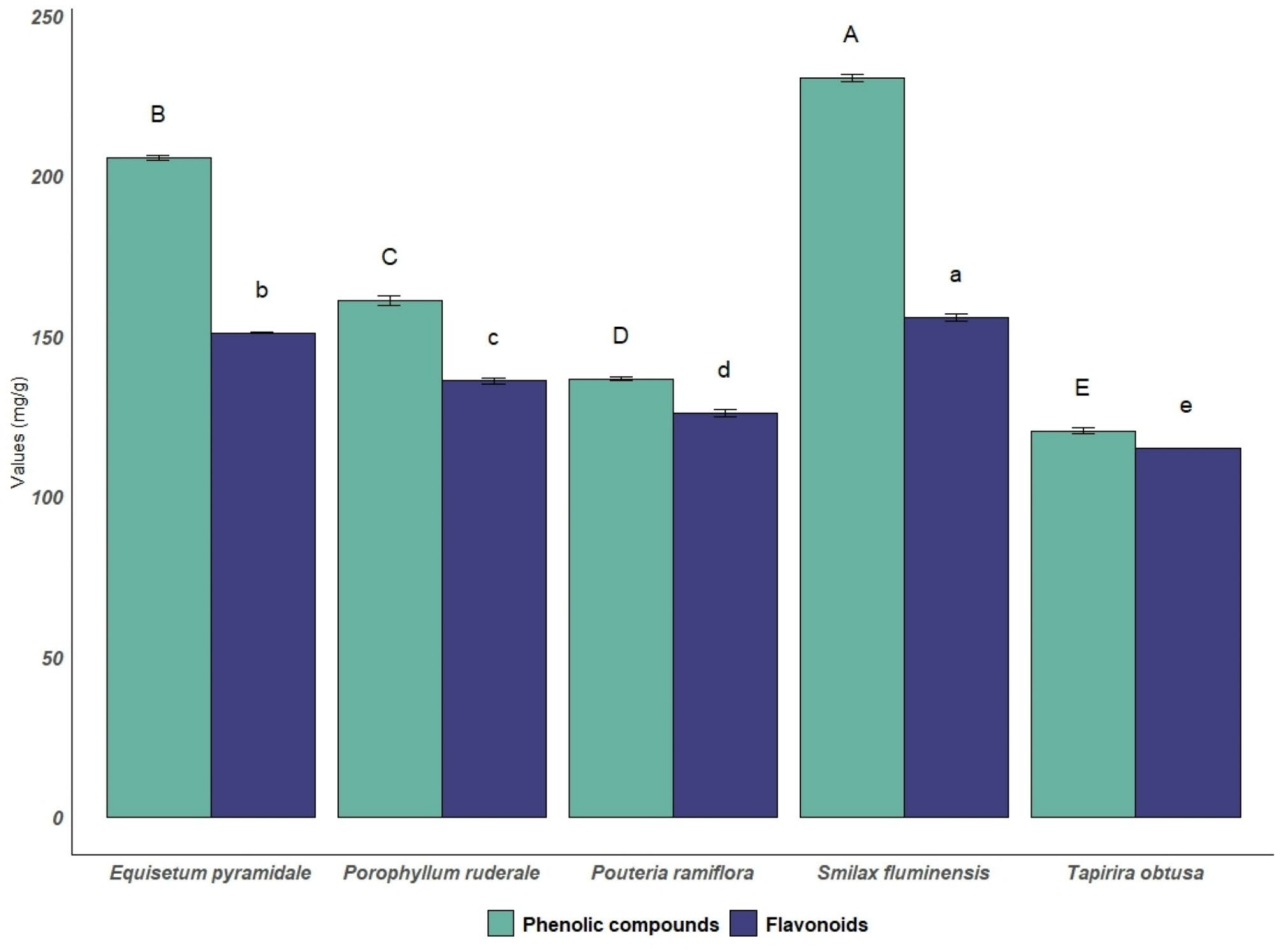
Phenolic compounds and flavonoids from *E. pyramidale*,* P. ruderale*,* P. ramiflora*,* S. fluminensis* and *T. obtusa*. Note: *Values ​​are expressed as the mean ± standard deviation (*n* = 3). The results of each test were analyzed separately by analysis of variance (ANOVA). Means with different letters in the same column are significantly different according to Tukey’s test, with *p* < 0.05. The capital letters indicate phenolic compounds, and the lowercase letters indicate flavonoids. **mg/g = milligrams of catechin/g of extract for phenolic compounds and mg quercetin/g extract for flavonoids

All extracts exhibited significant levels of phenolic compounds and flavonoids. The ethanolic extract of *S. fluminensis* had the highest content of phenolic compounds (230.34 ± 1.20 mg catechin/g extract) and flavonoids (155.77 ± 0.99 mg quercetin/g extract). The ethanolic extract of *E. pyramidale* contained phenolic compounds at 205.4 ± 0.70 mg catechin/g extract and flavonoids at 150.9 ± 0.25 mg quercetin/g extract. The results are presented in Fig. 3 and are expressed as the mean and standard deviation.

### Antioxidant activity

**Fig. 4:**
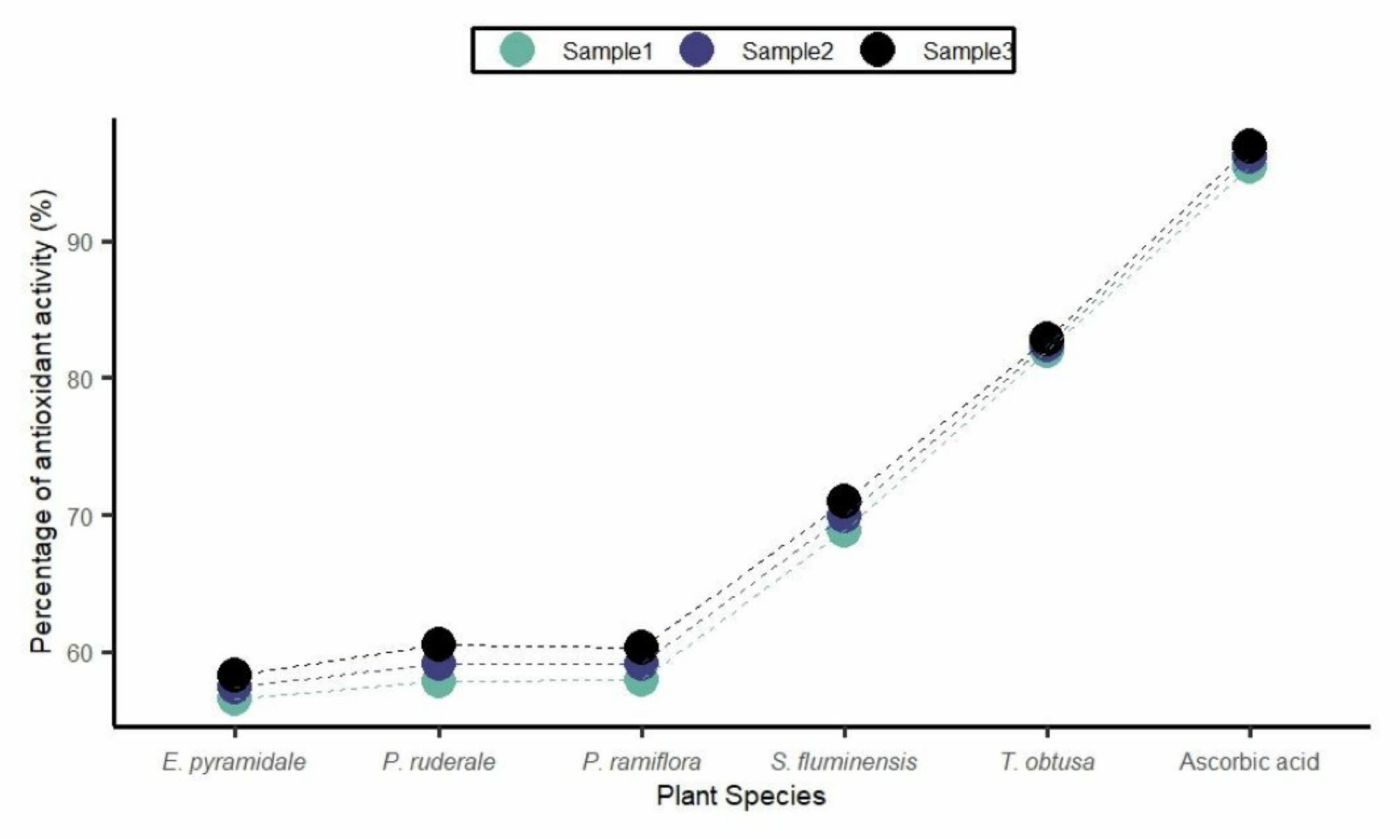
Percentage of antioxidant activity of plant extracts evaluated by free radical scavenging via the DPPH method. Note: The experiments were performed in triplicate, with sample 1, sample 2, and sample 3 corresponding to the individual replicates of each plant species tested

Figure 4 and Supplementary Table 1 illustrate the antioxidant activities of the evaluated plant species (*n* = 3 samples), expressed as the average percentage of DPPH free radical scavenging activity. The *T. obtusa* extract showed the highest antioxidant activity (82.36% ± 0.44), followed by *S. fluminensis* (69.89% ± 1.06).

### Antiproliferative activity and cytotoxicity

**Fig. 5:**
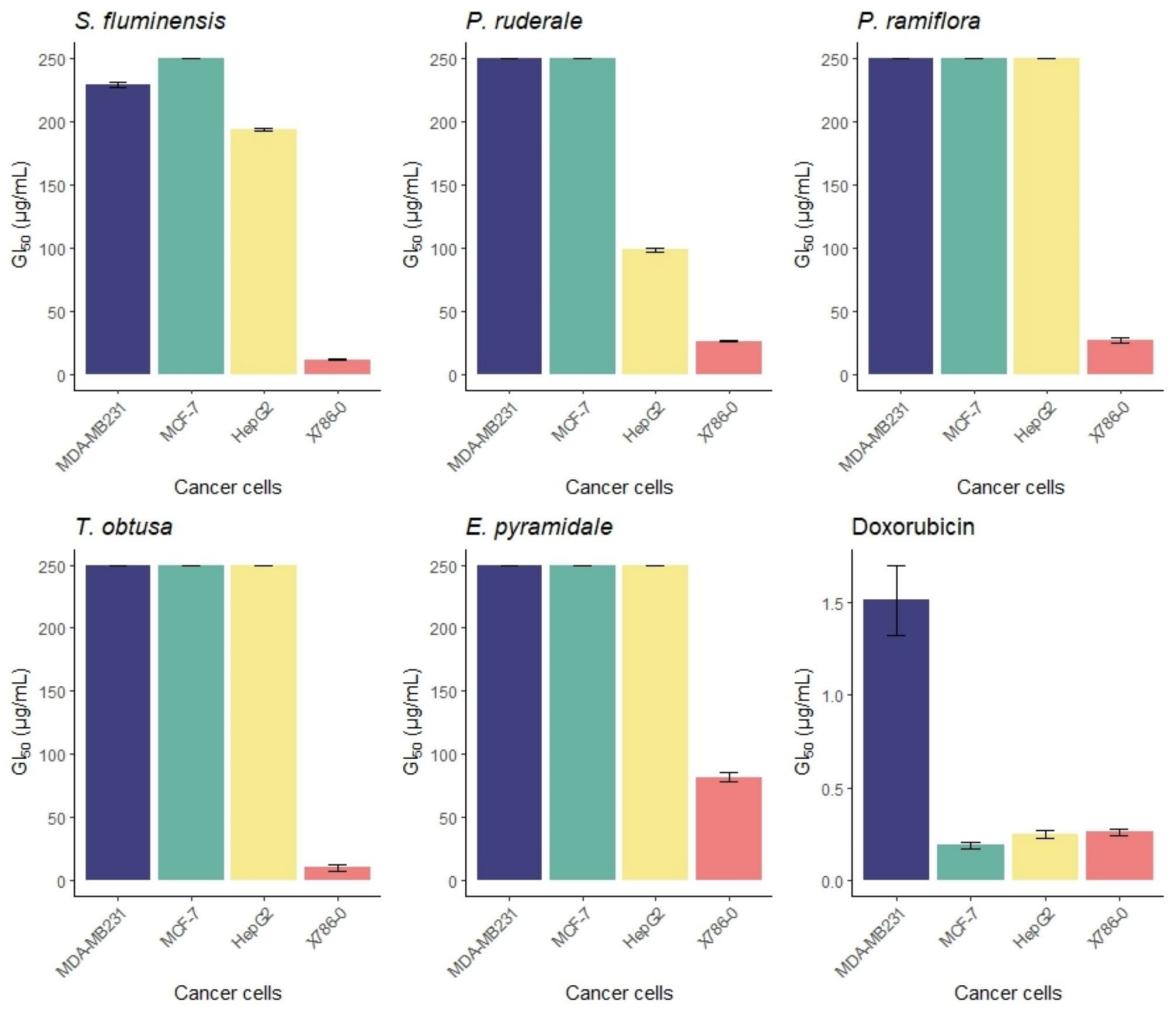
Antiproliferative effect of plant ethanolic extracts on human cancer cells (GI_50_ µg/mL). Note: Cancer cell lines: MDA-MB231 – multidrug resistant breast; MCF-7 breast with hormone receptor; 786-0 – kidney; HepG2– liver

The anti-proliferative activities of the five plant extracts against human breast, kidney, and liver neoplastic cells are shown in Fig. 5 and Supplementary Table 2. All tested extracts exhibited antiproliferative effects on neoplastic kidney cells within a 48 h treatment period. *P. ruderale* extracts also showed antiproliferative activity against liver neoplastic cells (GI50 98.33 ± 1.31 µg/mL).

Figure 5 illustrates the antiproliferative impact of the five plant extracts and doxorubicin, positive control, on human breast, kidney, and liver neoplastic cells. The results are expressed as the GI_50_, which is the effective concentration capable of inhibiting 50% of cell growth in vitro. The lower the GI_50_ value is, the more effective the compound, as a lower concentration is required to inhibit cell growth.

A cytotoxicity assay was performed to predict the in vitro safety of the plant extracts and assess their ability to interfere with cell viability. In this assay, the potential cellular toxicity of the five active plant extracts and doxorubicin was measured using the noncancerous fibroblastic cell line NIH/3T3. The results showed that the extracts of *P. ruderale*,* P. ramiflora*,* T. obtusa*, and *E. pyramidale* did not show toxicity up to the highest concentration tested (GI_50_ > 250 µg/mL), while the *S. fluminensis* extract showed a GI_50_ equal to 84.16 ± 2.21 µg/mL (Supplementary Table 2).

### Selectivity index

According to the selectivity analysis, all plant species exhibited high selectivity index (SI) values in the 786-0 cell line, ranging from 7.35 (*S. fluminensis*) to 24.61 (*T. obtusa*) (Table 1). The SI of *T. obtusa* was the highest among all the tested plants and cell lines, demonstrating its selective cytotoxicity against cancer cells of the 786-0 line compared to normal cells.

**Table 1:** GI_50_ (µg/mL) and SI values for the ethanolic extracts and doxorubicin (positive control)

Cell lines	*S. fluminensis*		*P. ruderale*		*P. ramiflora*		*T. obtusa*		*E. pyramidale*		Doxorubicin	
	GI_50_ (µg/mL)	SI	GI_50_ (µg/mL)	SI	GI_50_ (µg/mL)	SI	GI_50_ (µg/mL)	SI	GI_50_ (µg/mL)	SI	GI_50_ (µg/mL)	SI
MDA-MB231	228.72 ± 2.1	0.37	> 250	> 1	> 250	> 1	> 250	> 1	> 250	> 1	1.51 ± 0.19	1.56
MCF-7	> 250	> 0.34	> 250	> 1	> 250	> 1	> 250	> 1	> 250	> 1	0.19 ± 0.02	4.42
HepG2	193.5 ± 1.11	0.43	98 ± 1.31	3	> 250	> 1	> 250	> 1	> 250	> 1	0.25 ± 0.02	3.36
786-0	11.45 ± 0.83	7.35	26 ± 0.66	10	26.71 ± 1.87	9.36	10.16 ± 2.33	24.61	81.92 ± 3.34	3.05	0.26 ± 0.02	3.23
NIH_3T3	84.16 ± 2.21		> 250		> 250		250		> 250		0.84 ± 0.22	

Note: Cancer cell lines: MDA-MB231 – multidrug resistant breast; MCF-7 breast with hormone receptor; 786-0 – kidney; HepG2– liver. NIH/3T3: OECD-standardized murine fibroblast to assess cytotoxicity. SI: Selectivity index

### Antimicrobial susceptibility test

#### Disk diffusion method

The results of the antimicrobial susceptibility test using the disk diffusion technique with ethanolic extracts of the *E. pyramidale*,* P. ramiflora*,* P. ruderale*,* T. obtusa* and *S. fluminensis* against *C. albicans*, *C. parapsilosis*, *P. kudriavzevi*, *N. glabrata*, MRSA, and KP-KPC are shown in Table 2.

**Table 2:** Growth inhibition, in mm, of *Candida* spp., *Nakaseomyces glabrata*,* Pichia kudriavzevii*, MRSA, and KP-KPC by plant extracts via the disc diffusion method

Plant Species	C* µg/disc	Microorganisms					
		*Candida albicans* ATCC 90,028	*Nakaseomyces glabrata* ATCC 2001	*Pichia kudriavzevii* ATCC 6258	*Candida parapsilosis* ATCC 22,019	MRSA	KP-KPC
*E. pyramidale*	500	0	0	0	0	0	8.0 ± 0.0
	2000	0	0	0	0	9.0 ± 0.0	10.5 ± 0.71
*P. ruderale*	500	0	0	0	0	0	11.5 ± 0.71
	2000	0	0	0	0	0	13.5 ± 0.71
*P. ramiflora*	500	0	0	0	0	0	0
	2000	0	0	0	0	0	9.5 ± 0.71
*S. fluminensis*	500	0	11.0 ± 0.0	0	0	0	0
	2000	0	15.0 ± 0.0	0	0	8.0 ± 0,0	0
*T. obtusa*	500	0	0	0	0	0	0
	2000	0	0	0	0	8.5 ± 0.71	9.0 ± 1.41
Fluconazole^1^	25	17.33 ± 0.52	17.33 ± 0.52	0	17.33 ± 0.52	nt	nt
Chloramphenicol^2^	30	nt	nt	nt	nt	30.33 ± 1.37	nt
Ertapenem^3^	10	nt	nt	nt	nt	nt	9.50 ± 0.84
Distilled water^4^	-	0	0	0	0	0	0

MRSA = Methicillin-resistant *Staphylococcus aureus*. KP-KPC = *Klebsiella pneumoniae* carbapenemase-producing. The values are expressed as the means ± standard deviations (*n* = 2). * Concentration in µg/disc; 0 absence of activity (absent inhibition halo or < 8 mm). ^1^ quality control for yeast; ^2^ quality control for MRSA; ^3^quality control for KP-KPC; ^4^negative control; nt = not tested

In this screening, the *S. fluminensis* extract exhibited antimicrobial activity against *N. glabrata* at the two concentrations tested (500 µg/disc and 2,000 µg/disc). In the test carried out on MRSA, the extracts of *E. pyramidale*,* T. obtusa*, and *S. fluminensis* showed a growth inhibition halo only at the highest concentration (2,000 µg/disc). For KP-KPC, growth inhibition was observed around the disc of four plant extracts used: *P. ruderale*,* E. pyramidale*,* P. ramiflora* and *T. obtusa.*

#### Broth microdilution

The minimum inhibitory concentration (MIC) was determined for the ethanolic extracts that exhibited growth inhibition of 8 mm or more in diameter in the disc diffusion test.

The broth microdilution test with the ethanolic extract of *S. fluminensis* on *N. glabrata* showed an MIC equal to 500 µg/mL. When tested against MRSA, extracts of *T. obtusa* exhibited an MIC equal to 1,000 µg/mL. Conversely, however, an MIC greater than 2,000 µg/mL was found for the effects of the extracts of *E. pyramidale* and *S. fluminensis* on MRSA and those of *E. pyramidale*,* P. ruderale*,* P. ramiflora*, and *T. obtusa* on KP-KPC.

The *T. obtusa* extract exhibited bactericidal activity on MRSA at a concentration of 1,000 µg/mL, while the *S. fluminensis* extract demonstrated fungicidal activity on *N. glabrata* at a concentration of 500 µg/mL.

## Discussion

This study highlights the potential of *E. pyramidale*,* P. ramiflora*,* P. ruderale*,* T. obtusa*, and *S. fluminensis* as promising sources for the development of new therapies. The ethanol was selected as the extraction solvent due to its effectiveness in extracting polyphenols and its safety for human consumption [50].

We detected a high frequency of phenolic and flavonoid compounds in all the plants tested (*E. pyramidale*, *P. ramiflora*, *P. ruderale*, *S. fluminensis*, and *T. obtusa*). Previous studies have also reported a high frequency (100%) of phenolic compounds and flavonoids in the ethanolic extracts of *E. pyramidale* [24], *P. ramiflora* [27] and *S. fluminensis* [51]. However, this high frequency is not always observed. A study carried out by Corrêa et al. [52] reported a low frequency of flavonoids (25%) in ethanolic extract of *P. ramiflora* leaves.

Mendonça et al. [26] reported similar levels of phenolic compounds in the extract of *P. ruderale* but lower levels of phenolic compounds and flavonoids in *S. fluminensis* compared to our findings. In all the plants analyzed, phenolic compounds and flavonoids were the most prevalent, except for *T. obtusa* that exhibited a predominance of triterpenes, followed by phenolic compounds, corroborating previous findings [53]. These differences may stem from factors such as environmental conditions, collection time, plant parts analyzed, and extraction methods, all of which significantly influence secondary metabolite production [54]. Due to their pharmacological properties, phenolic compounds are widely recognized for their beneficial effects on human health, including on cardiovascular diseases [55, 56], neurodegenerative disorders [19, 57], cancer management [4, 57], antioxidants [19, 58], and anti-inflammatory treatment [56, 57].

Triterpenes are known to have anti-inflammatory [3], antiproliferative [59], antidiabetic [60], antimicrobial [61], analgesic [3], and immunomodulatory [62] effects.

In this study, all tested plant extracts exhibited antioxidant activity. To the best of our knowledge, this is the first description of the antioxidant activities of extracts from *P. ramiflora*,* T. obtusa*, and *E. pyramidale*. *Tapirira obtusa* has a high antioxidant potential, corroborating findings in another species of the genus, namely, *T. guianensis* [30]. Previous studies have verified the antioxidant activity of *P. ruderale* [63, 64].

The antioxidant activity detected in the extracts in this study may be related to flavonoids and triterpenes [4, 20, 58]. These compounds are known to interact and neutralize free radicals, reduce the formation of new reactive species, chelate metal ions that catalyze oxidation reactions, and influence cellular signaling pathways and gene expression related to oxidative stress [58, 65, 66]. Therefore, triterpenes may be associated with the antioxidant activity found in the extracts of *T. obtusa*, whereas flavonoids may be related to the antioxidant effects of other species.

The anticancer properties and therapeutic potential of secondary plant metabolites have been extensively studied. Our study is also the first to describe the antiproliferative activity of *T. obtusa* and *E. pyramidale* extracts. The results showed that all the extracts had potent activity against renal neoplastic cells, except for *E. pyramidale*, which exhibited moderate activity. In addition to its potent antiproliferative activity against renal neoplastic cells, the *P. ruderale* extract also exhibited moderate activity against hepatic neoplastic cells.

Previous studies have documented the antiproliferative effects of *P. ruderale*, *P. ramiflora*, and *S. fluminensis* on various cell lines. In vitro investigations have demonstrated the antiproliferative activity of *P. ramiflora* extract against human hepatocellular carcinoma cells (HepG2 cells) [67], as well as that of *P. ruderale* extract against human colon adenocarcinoma cells (SW480) [64]. Fetter et al. [52] demonstrated that an ethanolic extract of *S. fluminensis* exhibited antitumor activity both in vitro and in vivo, reducing tumor growth in B16-F10 cells.

The antiproliferative activity of the studied plants may be related to the presence of phenolic compounds and flavonoids [52, 67]. These compounds inhibit or slow the growth of cancer cells through the induction of apoptosis, inhibition of angiogenesis, and modulation of cell signaling pathways related to growth and cell division [68, 69]. Consequently, they contribute to cancer prevention.

In our study, no cytotoxicity was observed in non-tumor cells for the extracts of *E. pyramidale*, *P. ramiflora*, *P. ruderale*, or *T. obtusa*, suggesting that these extracts are promising for further comprehensive studies regarding their therapeutic safety and efficacy.

According to the selectivity analysis, all plant species exhibited high selectivity indices (SIs) in the 786-0 renal cancer cell line, ranging from 7.35 (*S. fluminensis*) to 24.61 (T. *obtusa*). *Tapirira obtusa* exhibited the highest SI among all tested plants and cell lines. Specifically, the SI in the 786-0 renal cancer cell line was significantly greater (SI = 24.61) than that in the control (SI = 3.23, doxorubicin), indicating its selective cytotoxicity against cancer cells and its potential as a therapeutic agent against renal cancer.

Although the extract of *S. fluminensis* demonstrated toxicity in murine fibroblasts, an SI of 7.35 suggested that the extract is more effective against cancerous cells (786-0) than against normal cells. This indicates a favorable selectivity profile, making the extract a candidate for therapeutic development. The results are preliminary, and therefore insufficient to establish molecular associations that explain this outcome. However, metastatic renal cell carcinoma is known to be particularly chemoresistant [70], to the extent that chemotherapy is not typically a first-line treatment. This resistance may be related to the high intracellular concentration of glutathione (GSH) in these cells, which protects them from the toxic effects of reactive oxygen species (ROS) and aids in the elimination of xenobiotics [71]. In this study, we identified that *T. obtusa* and *S. fluminensis*, the two species with the highest antioxidant activity, also showed the greatest antiproliferative effect against 786-0 cells. This correlation is significant and warrants further investigation.

Our results demonstrated that the ethanolic extract of *S. fluminensis* leaves has fungicidal activity against *C. glabrata* (ATCC 2001), currently known as *Nakaseomyces glabrata (N. glabrata)*. To our knowledge, this is the first documented investigation of the antifungal activity of *S. fluminensis* against *N. glabrata*. However, for a more comprehensive understanding, additional studies involving the testing of a larger number of *Candida* species need to be carried out. *N. glabrata* is the second most prevalent cause of vulvovaginal and oropharyngeal candidiasis [72]. This pathogen often exhibits reduced sensitivity to azoles and is resistant to echinocandins [73], thus highlighting the urgent need to develop new therapeutic approaches to treat candidiasis caused by this pathogen. *N. glabrata* is on the World Health Organization (WHO) list of priority fungal pathogens, emphasizing the need for research and policies focused on infections and antifungal resistance [74].

In addition to the current study, only one other documented study has investigated the antifungal activity of *S. fluminensis*. This previous study, conducted by Brito et al. [75], used extracts from the aerial parts and flowers of *S. fluminensis* and reported antifungal activity against *C. tropicalis* and *P. kudriavzevii*, previously known as *Candida krusei*. Other *Smilax* species have demonstrated antimicrobial activity in vitro [76–78].

This is the first study to demonstrate the antibacterial activity of *T. obtusa*. Our results revealed that the ethanolic extract of *T. obtusa* leaves exhibited antibacterial activity against MRSA. *Staphylococcus aureus* is the predominant etiological agent of skin and soft-tissue infections. In some regions, up to 60% of these infections are attributed to MRSA, representing a significant challenge for effective treatment, potentially leading to more severe cases and even death [79, 80]. This bacterium is listed by the WHO as a priority for new drug research and development [6].

The ethanolic extracts of *E. pyramidale*, *P. ramiflora*, *P. ruderale*, and *T. obtusa* exhibited inhibitory activity in the disk diffusion test against KP-KPC, although these results were not confirmed by broth microdilution testing at the concentrations tested. This could be explained by the lipopolysaccharide-rich hydrophilic surface of the cell wall of gram-negative bacteria, which hinders the diffusion of active compounds and acts as a barrier [81].

Differences in the methodologies used to assess antimicrobial activity can result in data that are difficult to compare between studies. The absence of specific methodologies for testing the antimicrobial susceptibility of plant extracts can lead to significant differences in results, which in turn may vary due to the culture medium used, inoculum preparation, and technique employed.

In addition to phenolic compounds and flavonoids, the antifungal activity of *S. fluminensis* may also be related to the presence of saponins and coumarins that exhibit antibacterial, antifungal, and antitumor activities [82].

The use of a single extraction solvent is one of the limitations of this study. Solvents with varying polarities could provide a more comprehensive profile of the bioactive compounds. Furthermore, future studies evaluating different plant parts and conducting collections in various seasons could help clarify the influence of these variables on the concentration of secondary metabolites and the activities observed. We also recommend increasing the number of pathogens isolates in antimicrobial assays and testing a wider range of cell lines to strengthen the analysis of the bioactivity and therapeutic potential of the plant extracts.

## Conclusions

The presence of phenolic and flavonoid compounds in E. *pyramidale*, P. *ramiflora*, *P. ruderale*, *T. obtusa* and *S. fluminensis* and triterpenes, in *T. obtusa*, may be related to the antioxidant and antiproliferative activities detected in the ethanolic extracts of these plants, which can be considered promising sources for the development of new drugs.

This study describes for the first time antioxidant activities in ethanolic extract of *P. ramiflora*, *T. obtusa* and *E. pyramidale* as well as antiproliferative activity in *T. obtusa* and *E. pyramidale*.

The results show that *P. ramiflora*,* P. ruderale*, *S. fluminensis* and *T. obtusa* extracts have potent antiproliferative effects on neoplastic kidney cells. *P. ruderale* extracts show antiproliferative activity against liver neoplastic cells.

Studies with a larger number of strains are necessary to confirm the antibacterial activity found in *T. obtusa* extract against MRSA and fungicidal activity in *S. fluminensis* extract against *N. glabrata*.

## Electronic supplementary material

Below is the link to the electronic supplementary material.

Supplementary Material 1

Supplementary Material 2

## Data Availability

The data supporting the findings of this study are provided within the manuscript and in supplementary files. The supplementary tables are attached in the files section under the titles ‘Supplementary Table 1’ and ‘Supplementary Table 2’, available as supplementary material.

## Abbreviations

ATCC: American type culture collection
*C. glabrata*: *Candida glabrata*
CLSI: Clinical laboratory standards institute
DPPH: 2,2 diphenyl-1-picrylhydrazyl
*E. pyramidale*: *Equisetum pyramidale golden*
FIOCRUZ: Oswaldo cruz foundation
GI_50_: Concentration that inhibits 50% of cell growth
HepG2 cells: Human hepatocellular carcinoma cells
KP-KPC: Carbapenemase-producing Klebsiella pneumoniae
MBC: Minimum bactericidal concentration
MFC: Minimum fungicidal concentration
MHA: Mueller-Hinton Agar
MIC: Minimum inhibitory concentration
MRSA: Methicillin-resistant Staphylococcus aureus
*N. glabrata*: *Nakaseomyces glabrata*
OECD: Organization for Economic Cooperation and Development
*P. kudriavzevi*: *Pichia kudriavzevii*
*P. ramiflora*: *Pouteria ramiflora* (mart.) *radlk*
*P. ruderale*: *Porophyllum ruderale jacq*
ROS: Reactive oxygen species
RPMI 1640: Roswell park memorial institute 1640
*S. fluminensis*: *Smilax fluminensis steud*
SDA: Sabouraud dextrose agar
SI: Selectivity index
SRB: Sulforhodamine B
SW480: Human colon adenocarcinoma cells
*T. guianensis*: *Tapirira guianensis*
*T. obtusa*: *Tapirira obtusa* (benth.) J.D. mitch
TCA: Trichloroacetic acid
WHO: World health organization
λmáx.: Maximum absorbance
