# Supplementary material for: Bioactives derived from Brazilian native flora with antimicrobial and anticancer activity
Source: BMC Complement Med Ther. 2025 Mar 11;25:102. doi: 10.1186/s12906-025-04787-0 (PMC11900633; doi:10.1186/s12906-025-04787-0)
Supplement: Supplementary file 1 — Supplementary Material 1 [file 12906_2025_4787_MOESM1_ESM.docx]

Supplementary Table 1 - Percentage of antioxidant activity by the DPPH method of plant extracts at a concentration of 50 mg/mL

| **Plant species** | **Antioxidant activity**  **(%) ± SD** |
| --- | --- |
| *E. pyramidale* | 57.43±0.86 |
| *P. ruderale* | 59.16±1.31 |
| *P. ramiflora* | 59.13±1.16 |
| *S. fluminensis* | 69.89±1.06 |
| *T. obtusa*  Ascorbic acid | 82.36±0.44  96.15±0.78 |

Supplementary Table 2 – Antiproliferative effect of plant ethanolic extracts on human cancer cells and non-tumor cells (GI_50_ μg/mL).

| Plant species | MDA-MB231  GI_50_ (μg/mL) | MCF-7  GI_50_ (μg/mL) | HepG2  GI_50_ (μg/mL) | 786-0  GI_50_ (μg/mL) | NIH/3T3  GI_50_(μg/mL) |
| --- | --- | --- | --- | --- | --- |
| *S. fluminensis* | 228.72±2.1 | >250 | 193.50±1.11 | 11.45±0.83 | 84.16±2.21 |
| *P. ruderale* | >250 | >250 | 98.33±1.31 | 26.02±0.66 | >250 |
| *P. ramiflora* | >250 | >250 | >250 | 26.71±1.87 | >250 |
| *T. obtusa* | >250 | >250 | >250 | 10.16±2.33 | >250 |
| *E. pyramidale* | >250 | >250 | >250 | 81.92±3.34 | >250 |
| Doxorubicin | 1.51±0.19 | 0.19±0.02 | 0.25±0.02 | 0.26±0.02 | 0.84±0.22 |

Note: Cancer cell lines: MDA-MB231 – multi-drug resistant breast; MCF-7 breast with hormone receptor; 786-0 – kidney; HepG2 – liver. NIH/3T3: OECD-standardized murine fibroblast to assess cytotoxicity.
