# Supplementary material for: Bioactives derived from Brazilian native flora with antimicrobial and anticancer activity
Source: BMC Complement Med Ther. 2025 Mar 11;25:102. doi: 10.1186/s12906-025-04787-0 (PMC11900633; doi:10.1186/s12906-025-04787-0)
Supplement: Supplementary file 2 — Supplementary Material 2 [file 12906_2025_4787_MOESM2_ESM.pdf]

To Marym Mohammad Hadi,

Editor of BMC Complementary Medicine and Therapies

Dear Dr:

Thank you for email regarding the technical check of my manuscript titled "BIOACTIVES DERIVED FROM BRAZILIAN NATIVE FLORA WITH ANTIMICROBIAL AND ANTI-CANCER ACTIVITY" (Manuscript ID: 0c69ca44-146f-4b7d-9acb-c2e3245016d5).

I would like to clarify that my study did not involve experiments using gels or blots (such as Western Blots). Therefore, we do not have images of these analyzes to provide as supplementary files. However, it is possible that I did not fully understand your request. If you could kindly clarify, I would be most grateful and will promptly address it.

Please let me know if further clarification or any other adjustments are needed for the submission to proceed smoothly.

Thank you again for your support and attention.

Best regards,

Marilene Rodrigues Chang
